# Differences in Adolescents’ Food Habits Checklist (AFHC) Scores before and during Pandemic in a Population-Based Sample: Polish Adolescents’ COVID-19 Experience (PLACE-19) Study

**DOI:** 10.3390/nu13051663

**Published:** 2021-05-14

**Authors:** Dominika Skolmowska, Dominika Głąbska, Dominika Guzek

**Affiliations:** 1 Department of Dietetics, Institute of Human Nutrition Sciences, Warsaw University of Life Sciences (SGGW-WULS), 159C Nowoursynowska Street, 02-776 Warsaw, Poland; dominika_skolmowska@sggw.edu.pl; 2 Department of Food Market and Consumer Research, Institute of Human Nutrition Sciences, Warsaw University of Life Sciences (SGGW-WULS), 159C Nowoursynowska Street, 02-776 Warsaw, Poland; dominika_guzek@sggw.edu.pl

**Keywords:** dietary habits, adolescents, national study, population-based study, Adolescents’ Food Habits Checklist (AFHC), Coronavirus-19, COVID-19, SARS-CoV-2, PLACE-19 Study

## Abstract

The COVID-19 pandemic is known to influence the dietary habits of adults, but results for adolescents in studies are ambiguous. The present work aimed to analyze the differences in the scores of the Adolescents’ Food Habits Checklist (AFHC) before and during the pandemic in the Polish Adolescents’ COVID-19 Experience (PLACE-19) Study population. The PLACE-19 Study was conducted during the pandemic among a population of 2448 students recruited from secondary schools in all regions of Poland using a random quota sampling. The participants were required to complete an AFHC consisting of 23 items pertaining to food purchase, preparation, and consumption habits. Current habits (during the pandemic) and previous habits were assessed and scored separately. The total (*p* = 0.001), purchase (*p* < 0.001), and consumption scores (*p* = 0.014) indicated that the AFHC scores during the pandemic were higher than before. For questions on purchase habits, a lower number of respondents reported eating in a restaurant, eating takeaway meals, having lunch away from home, or buying pastries, cakes or crisps. For questions on preparation habits, an greater number of respondents reported that they usually avoided eating fried food and tried to keep their overall sugar intake down, but fewer respondents said they tried to have low-fat desserts. For questions on consumption habits, a lower number of respondents reported that they usually ate a dessert or pudding if one were available and a larger number said they made sure to eat at least one serving of vegetables or salad a day and at least three servings of fruit most days. Based on the obtained results, it may be stated that although there was an increase in the AFHC scores during the pandemic, a similar share of respondents showed improved or worsened food habits, and a similar share changed their food habits from healthy to unhealthy and from unhealthy to healthy. At the same time, a majority of changes were associated with purchase habits, which were probably forced by lockdowns and the resultant restrictions in eating out or grocery shopping.

## 1. Introduction

In December 2019, a novel coronavirus, named Severe Acute Respiratory Syndrome Coronavirus 2 (SARS-CoV-2), emerged in Hubei Province, China, causing the respiratory disease COVID-19 [1]. The virus soon spread all over the world, and by March 2020, there were 96,000 confirmed COVID-19 cases worldwide [2]. Taking into consideration the global threat associated with the spread of this virus, the World Health Organization (WHO) declared COVID-19 a pandemic [3] and announced an international public health emergency [4]. To prevent the transmission of the virus, various protective measures were recommended, including strict social distancing, closure of schools and universities, home quarantine, isolation or lockdown, and the use of personal protective measures [5]. 

A widespread COVID-19 outbreak caused adverse mental health consequences, including stress, anxiety, and depression among the affected populations [6]. It is well established that chronic stress influences general health and may also change the health habits of the individuals, including their eating habits [7]. Prolonged stress induces the secretion of cortisol, which is a major component of stress response and increases hunger [8]. A number of studies on adolescents have indicated that stress is associated with increased intake of high-energy food rich in both sugar and fat [9,10]. A study conducted by Jeong & Kim [11] among South Korean middle school adolescents revealed that girls categorized under the high-stress group showed a significantly higher frequency of snacking and overeating than those categorized under the low-stress group. Similarly, the study of Cartwright et al. [12] proved that stress influences dietary practices in adolescents, as they reported decreased consumption of fruit and vegetables and increased consumption of snacks and other fatty foods. These suggest that the COVID-19 pandemic may be associated with prolonged stress and thus result in relevant changes in dietary habits [13]. 

Moreover, diet and the resultant nutritional status of individuals have a substantial effect on the immune system and may influence susceptibility to disease [14]. In particular, malnutrition [15], as well as protein and micronutrient deficiencies lead to poor immune functions [16]. Therefore, adopting healthy dietary habits to help maintaining proper nutrition is crucial especially during the pandemic [17,18]. 

Another issue influencing the dietary habits of people is limited access to daily grocery shopping due to lockdowns, which may decrease the eating of fresh food (e.g., vegetables, fruit and fish) and increase the consumption of processed food [19]. Last but not least, some people may experience job loss, salary reduction, and general economic insecurity because of the pandemic, and as a result may limit their food expenses, which becomes a barrier to following healthy dietary habits [20].

A recent study by Sidor & Rzymski [21], conducted in the Polish population, confirmed that lockdowns may constitute a major dietary risk factor, as some people may display improper habits, characterized by a low intake of fruit and vegetables and a high intake of sweets. Similarly, the study of Carroll et al. [22], conducted among Canadian families, revealed that a majority of respondents consumed snacks more often during the pandemic than before. In addition, it indicated that children and adolescents showed an increased consumption of highly processed, calorie-dense comfort food, which may contribute to excessive weight gain [23]. This was confirmed in a study conducted among Italian adolescents that showed lower age groups increasing their consumption of junk food such as packaged sweets, baked products, sweet beverages, and savory snacks [19]. On the other hand, some authors reported beneficial changes in dietary habits during the pandemic, as adolescents in Spain, Italy, Brazil, Chile, and Colombia significantly increased their consumption of vegetables, fruit, and legumes [24].

Taking into account the contradictory results shown by the studies on dietary habits of adolescents, the present study aimed to analyze the differences in the scores on the Adolescents’ Food Habits Checklist (AFHC) scores before and during the pandemic in the Polish Adolescents’ COVID-19 Experience (PLACE-19) Study.

## 2. Materials and Methods

### 2.1. Ethical Statement

The study was conducted at the Institute of Human Nutrition Sciences, Warsaw University of Life Sciences (WULS-SGGW) in accordance with the guidelines laid down in the Declaration of Helsinki. All procedures involving human subjects received the approval of the Ethics Committee of the Institute of Human Nutrition Sciences of the Warsaw University of Life Sciences, as presented previously [25].

### 2.2. Studied Population

This study was carried out in a population-based sample of Polish secondary school students within the second phase of the national PLACE-19 Study. The adolescents who participated in the study were aged 15–20, which is a typical range for Polish secondary school students, for whom the Net Enrolment Rate (NER) is 89.38% [26]. The first phase of the study was carried out in April 2020 focusing on hand washing and personal protective behavior during this period [27,28]. The second phase was conducted in May 2020 (first stage—from 29 April to 10 May, and second stage—from 11 to 23 May), and focused on dietary habits [29]. 

During the study period, in accordance with the decision of the Polish Ministry of Education, made on 12 March 2020 [30], education in primary and secondary schools, as well as universities, was suspended, and a system of remote learning was implemented for all students. Other restrictive measures implemented in the country included the following: border sanitary control (since 9 March 2020), cancellation of mass events (since 10 March 2020), closure of cultural institutions (since 12 March 2020), and temporary closure of borders to noncitizens (since 15 March 2020) [31]. At the same time, since 14 March 2020, restaurants were allowed to provide food only for takeaway and delivery. In addition, only grocery stores, pharmacies, and laundry facilities remained open in shopping malls. Entertainment and recreation facilities were suspended, and public gatherings of more than 50 people were banned [31].

To obtain a national sample of adolescents from all six regions of Poland (defined by geography and socioeconomic condition), a stratified sampling of secondary schools was used: (1) a random selection of five counties from each of the 16 voivodeships (provinces) of Poland for 80 counties in total, and (2) a random selection of secondary schools: ten from each of the 80 counties for 800 schools in total during the first phase. In the case of voivodeships for which a low response rate was observed after 10 days of data collection during this stage, an identical sampling procedure was conducted involving: (1) a random selection of counties (five from each of the eight voivodeships for 40 counties in total) and (2) a random selection of secondary schools within the counties (10 secondary schools from each of the 40 counties) for a total of 400 schools during the second phase. 

A total of 1200 secondary schools were randomly selected and invited to participate in the study. The first phase (carried out in April 2020) assessed hand washing and personal protective behavior) and the schools that participated were excluded from the second phase. If necessary, the local boards of education were involved in procedural issues. 

The headmaster of each randomly selected secondary school received an invitation for the school to take part in the study and was informed about its aim and scope. As participation was voluntary, the headmaster forwarded the invitation to the students. Those who were willing to take part were required to provide written informed consent, as well as the consent of their parents or legal guardians. The inclusion criteria for students included the following: being a student from a randomly chosen school, 15–20 years of age, and providing informed consent. 

As soon as consent was obtained, the students received an electronic link to the questionnaire. The dedicated questionnaire was anonymous and did not collect any data that could identify the respondent. It also did not have any questions concerning personal or sensitive data except for age and the secondary school the student attended to verify the inclusion criteria. Because some of the headmasters who received the invitation did not report if they were interested in participating in the study, a reminder was sent after 1 week of the first stage and after 1 week of the second stage. 

Before the students completed the questionnaire, they were informed that the received link to the survey was dedicated only to them and could not be passed on to any other student because they constitute a specific study group and were chosen exclusively to take part in the study. 

**Figure 1:**
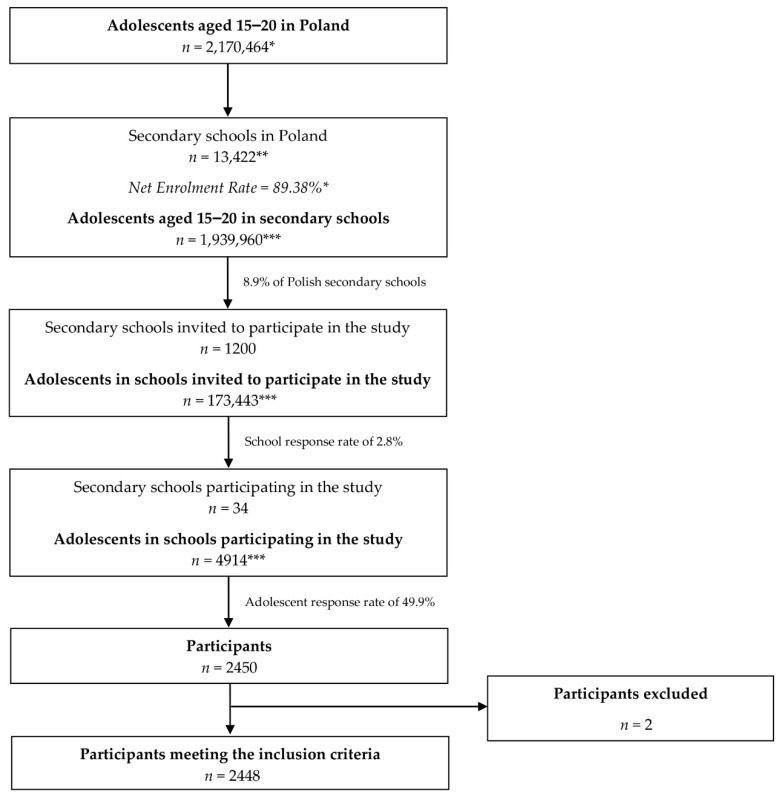
The procedure of secondary school sampling and students including to the PLACE-19 Study. * data by the Central Statistical Office (CSO) in Poland [26,32]; ** data by the Polish Ministry of National Education [33]; *** on the basis of the data by CSO.

After the students completed the questionnaire, the answers were verified for the presence of missing or unreliable data, and the respondents who provided such data were excluded from the analysis (Figure 1). 

### 2.3. Adolescents’ Food Habits Checklist (AFHC)

The questionnaire used in the study to collect information about dietary habits during the COVID-19 pandemic was AFHC, which was developed by Johnson et al. [34] and validated in a group of adolescents [34]. It is a commonly applied and efficient tool that enables, the eating habits of not only adolescents [35,36] but also adults [37] to be determined.

The participants were asked about their dietary habits before and during the pandemic. As remote learning had been implemented for all students in Poland, according to the decision of the Polish Ministry of National Education [30], the students were asked to focus on the period of remote learning (to serve as an easily recognizable point in time), and the questions were formulated to analyze dietary habits before and during remote learning.

As there was no Polish version of AFHC available before the study, it was translated into Polish based on the WHO recommendations [38]. Translation into Polish (forward translation) was conducted by a native Polish speaker, a researcher who was familiar with the discipline, to maintain semantic, idiomatic, cultural, and conceptual equivalence. The forward translation was followed by a backward translation into English, which was conducted by an independent translator who had no knowledge of the questionnaire. Finally, the questionnaire was analyzed by an expert panel of native Polish researchers who were fluent in English and did any polishing that was needed.

The applied AFHC consists of questions concerning issues associated with purchasing, preparing, and consuming certain types of food, both healthy and unhealthy. The highest possible score was 23 points, as described by Johnson et al. [34]. Additional analysis was conducted in the same way, but separately for purchase, preparation, and consumption habits, and the scores were calculated for 6, 8, and 9 related items, respectively. Moreover, for each respondent, dietary habits were classified under the categories healthy, neutral, and unhealthy, based on the quartile distribution, as applied by Proserpio et al. [35], with the first quartile attributed to unhealthy behavior, the second and third to neutral behavior, and the fourth to healthy behavior.

### 2.4. Statistical Analysis

The internal reliability of the data was assessed using the Cronbach’s alpha coefficient [39] which was interpreted based on a commonly applied criteria [40]. The normality of distribution of data was verified using Shapiro–Wilk test. The results were compared using the Chi Square test and the Mann–Whitney *U* test with a rank-biserial correlation (due to nonparametric distribution).

The Confirmatory Factor Analysis (CFA) was performed for internal reliability assessment. The Comparative Fit Index (CFI), Root Mean Square Error of Approximation (RMSEA), and Standardized Root Mean Square Residual (SRMR) were calculated. A similar approach was applied in the previously published paper with following cutoff criteria: CFI ≥ 0.90, RMSEA ≤ 0.06 (good fit), and SRMR ≤ 0.08 [41]. The CFA presented a good model fit (independently of the pandemic), with RMSEA = 0.058 (95% confidence interval (CI: 0.056, 0.061)), CFI = 0.926 and SRMR = 0.055.

The Cronbach’s alpha coefficient for dietary habit scores within the PLACE-19 Study population before and during the pandemic were calculated on the basis of the AFHC for purchase, preparation, consumption and total habits and are presented in Table 1. Based on the Cronbach’s alpha coefficient for the AFHC total score, a good internal reliability was stated, while for the purchase, preparation and consumption score, a good or acceptable internal reliability was observed. The highest score was for the AFHC-consumption score.

The value of *p* ≤ 0.05 was attributed to statistically significant differences. The statistical analysis was conducted using Statistica 13.3 (StatSoft Inc., Tulsa, OK, USA).

## 3. Results

The food purchase dietary habits assessed on the basis of the AFHC by Johnson et al. [34] within the PLACE-19 Study population are presented in Table 2. It was stated that while comparing the reported food purchase dietary habits during and before the pandemic, there were statistically significant differences for a majority of items. During the pandemic, the number of respondents who often bought pastries or cakes decreased (21.9% vs. 33.0%; *p* < 0.001) but the number of respondents who rarely ate takeaway meals increased (82.0% vs. 76.1%; *p* < 0.001). At the same time, for having lunch away from home, the number of both respondents choosing or not choosing a low-fat option decreased, while the number of those not having lunch away from home increased (40.3% vs. 21.5%, *p* < 0.001). However, for buying crisps and having a dessert or pudding in a restaurant, the number of respondents who reported choosing a low-fat crisps brand or the healthiest dessert or pudding did not change; only the number of those who declared not doing it decreased, whereas the number of respondents who declared not buying crisps (24.7% vs. 19.7%, *p* = 0.001) or not eating in a restaurant increased (39.2% vs. 25.7%, *p* < 0.001).

The food preparation dietary habits assessed on the basis of the AFHC by Johnson et al. [34] within the PLACE-19 Study population for the periods before and during the COVID-19 pandemic are presented in Table 3. It was stated that while comparing the declared food preparation dietary habits for these periods, there were statistically significant differences for some items. During the pandemic, compared with the period before, the number of respondents who usually avoided eating fried foods increased (32.8% vs. 24.5%; *p* < 0.001) as did the number of respondents trying to keep their overall sugar intake down (58.2% vs. 54.9%; *p* = 0.023). At the same time, regarding dessert at home, the number of respondents not trying to have something low in fat decreased (57.3% vs. 60.4%, *p* = 0.018), as they either started to try to have something low in fat, or avoided having desserts at home at all.

The food consumption dietary habits assessed on the basis of the AFHC by Johnson et al. [34] within the PLACE-19 Study population for the periods before and during the pandemic are presented in Table 4. It was stated that while compared the declared food consumption dietary habits for these periods, some items showed statistically significant differences. During the pandemic, compared with the period before, the number of respondents usually eating a dessert or pudding if one were available decreased (69.2% vs. 72.6%; *p* = 0.009). At the same time, the number of respondents making sure to eat at least one serving of vegetables or salad a day increased (69.5% vs. 66.5%; *p* = 0.027) as did the number of respondents eating at least three servings of fruit most days (42.8% vs. 39.5%; *p* = 0.024).

The dietary habits scores calculated on the basis of the AFHC by Johnson et al. [34] for purchase, preparation, consumption and total habits, within the PLACE-19 Study population before and during the pandemic are presented in Table 5. The AFHC scores observed for groups differed: during the pandemic, a higher total score (*p* = 0.001), purchase score (*p* < 0.001) and consumption score (*p* = 0.014) were observed.

Changes in dietary habit scores for the purchase, preparation, consumption of food, as well as total habits in the categories of healthy, neutral and unhealthy habits (calculated on the basis of the AFHC by Johnson et al. [34] within the PLACE-19 Study) are presented in Table 6. While comparing the categories of healthy, neutral and unhealthy habits, the majority of respondents for each score presented unchanged habits (ranging from a 66.3% purchase score to a 78.7% total score). The share of respondents presenting improved and worsened habits in healthy, neutral and unhealthy habits was similar (10.7−17.0%, improved; 10.6−16.7%, worsened).

## 4. Discussion

### 4.1. AFHC Scores in Other Studies

To our knowledge, this is the first study to assess the dietary habits of a population-based national sample of adolescents during the pandemic, using the AFHC, which is a reliable tool [34] for determining the dietary habits of different populations, including adolescents, and has been used in a number of studies [34,35,36,42,43]. 

In the present study, the median total AFHC score determined during the pandemic was 13.32 and before the pandemic was 12.65, while in most of the studies using AFHC, the total scores were lower. The study of Viggiano et al. [36] conducted in a group of Italian children and adolescents indicated that an AFHC score obtained without any intervention was 10.9. Similarly, the study by Kalkan [37] carried out among Turkish adolescents assessing their eating habits using AFHC revealed that females obtained significantly higher scores than males (mean score: 10.37 vs 9.26, respectively), and thus inferred that women were having more health-promoting eating habits than men. Interesting findings were also observed by Koca and Arkan [42] in their study determining the dietary habits of Turkish adolescents. The authors reported that the AFHC score was significantly lower in adolescents who did not perform any sport (9.26) compared to those involved in some sport (10.36), and in adolescents who skipped meals (9.33) compared to those who did not skip any (10.33). Moreover, the study on dietary habits by Chung et al. [43] indicated that Korean female adolescents earned an average AFHC score of 10.4. Thus, it can be emphasized that almost all the indicated AFHC scores are lower than those obtained in the present study. 

### 4.2. Potential Influence of the COVID-19 Pandemic on AFHC Scores

The high AFHC scores observed in the present study may be attributed to the fact that the study was conducted during the COVID-19 pandemic, which is said to be associated with changing food priorities [44]. Moreover, in our previous study of a population of Polish adolescents, it was observed that the pandemic changed food choice determinants, as participants reported an increase in the importance given to health and weight control and a reduction in the importance given to mood and sensory appeal [29]. Such positive changes in food choice determinants may positively influence food habits, but they may also influence the declaration from the previous period, thus indicating the possibility of recall bias.

At the same time, it must be specified that although there was a change in the median AFHC score obtained by the studied group, the classification of food habits did not change for a majority of respondents, and those who had healthy food habits before the pandemic followed them during it, while those who had unhealthy food habits before the pandemic still followed them. Similarly, the share of respondents who changed food habits from healthy to unhealthy and from unhealthy to healthy was almost the same, indicating that changes observed for the general population were not translated into improved food habits for individuals. Depending on the subgroup, food habits either improved or worsened. For adult populations, similar results were observed as some authors indicated that, depending on the subgroup, dietary behavior in the assessed populations during the pandemic either improved or worsened. For instance, such a result was observed by Deschasaux-Tanguy et al. [45] in a population of 37,252 French adults from the NutriNet-Sante cohort, and by Scarmozzino and Visioli [20] in a population of 1932 Italian adults.

It should also be mentioned that while the pandemic may have imposed a new challenge to follow a healthy diet, the declaration of food habits indicated that adolescents made some changes to their dietary habits. In the present study, changes were associated with food purchase. Adolescents were less likely to eat takeaway meals or have lunch away from home and more likely to avoid eating in a restaurant than before. This may be associated with the fact that restaurants were only allowed to offer food for takeaway and delivery in accordance with the decision of the Polish government [46]. Another important factor that may have influenced the decision not to have lunch away from home was the fear of social gatherings and the possibility of virus infection. This corresponded with the results observed in the study by Hayward et al. [47], who revealed that eating in restaurants was associated with higher odds of contracting an acute respiratory infection than traveling on a bus.

Regarding food habits, it must be emphasized that reducing the frequency of eating takeaway meals and avoiding eating in restaurants may have beneficial effects on dietary practices, for both adults and children. A study by Laguna et al. [44] conducted in the Spanish population showed that during lockdown an increasing number of respondents declared a higher frequency of cooking at home to entertain their children. Moreover, a report on the influence of COVID-19 on American’s food habits pointed out that 51% of respondents declared that they would continue to cook at home more even after the pandemic [48]. It may also be supposed that adolescents may benefit from a higher frequency of home cooking, as they may learn additional skills, including those that may improve their nutritional knowledge [49]. In addition, reducing the frequency of having lunch away from home allows a family to incorporate meals into a routine [50] as a study showed that adolescents who often eat alone are more likely to be overweight or obese [51]. 

However, it must be noted that changes in food habits during the pandemic, either due to legal regulations (for restaurants takeaway and delivery food only) or fear of infection, were not influenced by a conscious decision to improve the diet, but by the lack of possibility of following the same diet as before. As a result, even if the respondents declared that they would follow a healthier diet even after the pandemic, as in the study by Hunter [48], we do not know if they would revert to previous unhealthy habits.

The present study also revealed some relevant findings about specific purchase habits: the number of respondents who often bought pastries or cakes was lower during the pandemic than before. Similar results were also obtained for buying crisps, as there was a significant decrease in the number of respondents who declared buying crisps during COVID-19. Such dietary changes may be directly associated with a range of restrictions imposed by the Polish government, including restrictions on restaurants [46] and the number of customers permitted in shops [52], separate shopping hours for seniors [49], and general limitations on going out [53]. The study of Rodríguez-Pérez et al. [54] also showed that the lockdown led to the adoption of healthier dietary habits by the Spanish population, as a higher adherence to the Mediterranean diet was observed with a simultaneous decrease in the intake of snacks, fast food, fried food, pastries, and sweet beverages, which is similar to the changes in dietary choices observed in the present study. 

However, the study also revealed some beneficial consumption habits that were not directly associated with pandemic restrictions, but might probably have resulted from a conscious decision to change diet. It was found that a lower number of respondents avoided eating fried food and a higher number tried to reduce sugar intake compared to the period before the pandemic. Additionally, respondents were less likely to eat a dessert or pudding, even if it were available. 

Such changes observed in the studied group of adolescents may have especially favorable effects, as they comply with the recommendations of the Food and Agriculture Organization of the United Nations (FAO) for maintaining a healthy diet, which emphasize the need to reduce the intake of fat, sugar, and salt [55]. Because COVID-19 is associated with fear, anxiety, and despair [56], it can adversely affect mental health and that stress may trigger overeating and a desire to consume certain foods, particularly sweet ones [57].

Moreover, the present study showed that adolescents were more likely to eat at least one serving of vegetables or salad a day and at least three servings of fruit most days. These results are consistent with those obtained in the study by López-Bueno et al. [58], which pointed out that during lockdown a decreased share of people declared eating fewer than three servings of vegetables or fruit a day. It must be indicated that if a cognizant decision to change the food habits is made, then there is a chance that a healthy lifelong diet will be followed; if not, it will probably be only a temporary enhancement. 

On the other hand, some authors stated that the COVID-19 period promoted negative changes in eating habits in the studied populations [19,20,21,59]. Therefore, even if it is not proven that the observed changes will become stable, the positive changes observed in the food habits of Polish adolescents in the present study must be considered beneficial. However, to promote such positive changes, adequate nutritional education is necessary.

### 4.3. Strengths and Limitations of the Study

One of the strengths of the present study is that it was conducted in a large homogenous sample of adolescents recruited from all regions of Poland. In addition, it was conducted during a specific period of the pandemic, and provided valuable information regarding changes to dietary behavior, which had not been studied. Moreover, the study not only indicated the frequency of specific kinds of behavior but also made possible an assessment of the changes at the population level, revealing a similar share of beneficial dietary habits during the pandemic as in the period before.

The study also has some limitations that must be indicated. The use of AFHC to assess dietary habits is the first because the information obtained is based on self-reporting. A retrospective analysis was carried out simultaneously, but it was associated with recall bias. Furthermore, some of the changes in the assessed dietary behaviors reported by the respondents may not have been conscious choices but imposed by the general situation in Poland, including the lockdown and remote education. 

Taking into account the above limitations of the study, the necessary areas that should be focused on in further studies should be indicated. The pandemic is still influencing the lifestyle of populations, so there is a need for prospective studies of dietary habits. In the initial phase of the pandemic, when the situation was unpredictable, only retrospective studies were possible, but now it is possible to observe changes in the whole population and relate them to the current epidemiologic situation. Therefore, such studies should be conducted in various countries and the potential differences among populations observed. Last but not least, the studies should consider the influencing factors to indicate the determinants of dietary habit changes during the pandemic. 

## 5. Conclusions

Although an increase in AFHC scores was observed in the studied group during the COVID-19 pandemic, the study revealed that a similar share of respondents adopted improved and worsened food habits, and a similar share changed their food habits from healthy to unhealthy and from unhealthy to healthy. At the same time, a majority of changes were associated with purchasing habits, which were probably forced by the lockdown and resultant restrictions in eating out or grocery shopping. Taking these into account, it may be concluded that healthy food habits should be promoted among adolescents, especially during the pandemic, as some positive changes observed in this period may have been caused by existing limitations, and afterwards previous unhealthy habits may be revived or sustained.

## Figures and Tables

**Table 1:** The Cronbach’s alpha coefficient for dietary habits scores calculated on the basis of the AFHC for purchase, preparation, consumption and total habits, within the PLACE-19 Study.

AFHC Scores *	Before the COVID-19 Pandemic	During the COVID-19 Pandemic
AFHC total score	0.83	0.85
AFHC purchase score	0.61	0.63
AFHC preparation score	0.64	0.63
AFHC consumption score	0.75	0.77

* AFHC (Adolescents’ Food Habits Checklist) statements as described by Johnson et al. [34].

**Table 2:** The food purchase dietary habits assessed on the basis of the Adolescents’ Food Habits Checklist (AFHC) within the PLACE-19 Study.

Declared Habits Based on AFHC *		Before the COVID-19 Pandemic	During the COVID-19 Pandemic	*p* **
Choosing a low-fat option while having lunch away from home	Confirmed	854 (34.9%)	607 (24.8%)	<0.001
	Denied	1067 (43.6%)	854 (34.9%)	
	Not applicable	527 (21.5%)	987 (40.3%)	
Choosing a low-fat brand while buying crisps	Confirmed	420 (17.1%)	429 (17.5%)	0.001
	Denied	1546 (63.2%)	1415 (57.8%)	
	Not applicable	482 (19.7%)	604 (24.7%)	
Often buying pastries/cakes	Confirmed	808 (33.0%)	536 (21.9%)	<0.001
	Denied	1640 (67.0%)	1912 (78.1%)	
Rarely eating takeaway meals	Confirmed	1862 (76.1%)	2008 (82.0%)	<0.001
	Denied	586 (23.9%)	440 (18.0%)	
Usually choosing a diet option while buying a soft drink	Confirmed	899 (36.7%)	943 (38.5%)	0.194
	Denied	1549 (63.3%)	1505 (61.5%)	
Usually choosing the healthiest option while having a dessert/pudding in a restaurant	Confirmed	403 (16.5%)	375 (15.3%)	<0.001
	Denied	1416 (57.8%)	1114 (45.5%)	
	Not applicable	629 (25.7%)	959 (39.2%)	

* AFHC (Adolescents’ Food Habits Checklist) as described by Johnson et al. [34]; ** Chi Square test.

**Table 3:** The food preparation dietary habits assessed on the basis of the Adolescents’ Food Habits Checklist (AFHC) within the PLACE-19 Study.

Declared Habits Based on AFHC *		Before the COVID-19 Pandemic	During the COVID-19 Pandemic	*p* **
Usually avoiding eating fried foods	Confirmed	599 (24.5%)	803 (32.8%)	<0.001
	Denied	1849 (75.5%)	1645 (67.2%)	
Trying to keep the overall fat intake down	Confirmed	1302 (53.2%)	1299 (53.1%)	0.933
	Denied	1146 (46.8%)	1149 (46.9%)	
Trying to keep the overall sugar intake down	Confirmed	1345 (54.9%)	1425 (58.2%)	0.023
	Denied	1103 (45.1%)	1023 (41.8%)	
Trying to have something low in fat while having a dessert at home	Confirmed	701 (28.6%)	718 (29.3%)	0.018
	Denied	1479 (60.4%)	1403 (57.3%)	
	Not applicable	268 (11.0)	327 (13.4%)	
Usually eating at least one serving of vegetables/salad with the evening meal	Confirmed	1768 (72.2%)	1822 (74.4%)	0.087
	Denied	680 (27.8%)	626 (25.6%)	
Usually spreading butter/margarine on bread thinly	Confirmed	1607 (65.6%)	1570 (64.1%)	0.402
	Denied	521 (21.3%)	528 (21.6%)	
	Not applicable	320 (13.1%)	350 (14.3%)	
Usually including some chocolate and/or biscuits while having a packed lunch	Confirmed	601 (24.6%)	440 (18.0%)	0.638
	Denied	1536 (62.7%)	1083 (44.2%)	
	Not applicable	311 (12.7%)	925 (37.8%)	
Often having cream on desserts	Confirmed	434 (17.7%)	436 (17.8%)	0.248
	Denied	1604 (65.6%)	1559 (63.7%)	
	Not applicable	410 (16.7%)	453 (18.5%)	

* AFHC (Adolescents’ Food Habits Checklist) as described by Johnson et al. [34]; ** Chi Square test.

**Table 4:** The food consumption dietary habits assessed on the basis of the Adolescents’ Food Habits Checklist (AFHC) within the PLACE-19 Study.

Declared Habits Based on AFHC *		Before the COVID-19 Pandemic	During the COVID-19 Pandemic	*p* **
Usually eating a dessert/pudding if there is one available	Confirmed	1778 (72.6%)	1694 (69.2%)	0.009
	Denied	670 (27.4%)	754 (30.8%)	
Making sure that they eat at least one serving of fruit a day	Confirmed	1758 (71.8%)	1776 (72.5%)	0.588
	Denied	690 (28.2%)	672 (27.5%)	
Avoiding eating lots of sausages/burgers	Confirmed	1399 (57.2%)	1339 (54.7%)	0.920
	Denied	774 (31.6%)	747 (30.5%)	
	Not applicable	275 (11.2%)	362 (14.8%)	
Making sure that they eat at least one serving of vegetables/salad a day	Confirmed	1629 (66.5%)	1702 (69.5%)	0.027
	Denied	819 (33.5)	746 (30.5%)	
Trying to ensure that they eat plenty of fruit/vegetables	Confirmed	1800 (73.5%)	1748 (71.4%)	0.103
	Denied	648 (26.5%)	700 (28.6%)	
Often eating sweet snacks between meals	Confirmed	1149 (46.9%)	1076 (44.0%)	0.103
	Denied	1299 (53.1%)	1372 (56.0%)	
Often choosing fruit while having a snack between meals	Confirmed	1255 (51.3%)	1328 (54.3%)	0.075
	Denied	968 (39.5%)	892 (36.4%)	
	Not applicable	225 (9.2%)	228 (9.3%)	
Most days eating at least 3 servings of fruit	Confirmed	968 (39.5%)	1047 (42.8%)	0.024
	Denied	1480 (60.5%)	1401 (57.2%)	
Generally trying to have a healthy diet	Confirmed	1745 (71.3%)	1772 (72.4%)	0.409
	Denied	703 (28.7%)	676 (27.6%)	

* AFHC (Adolescents’ Food Habits Checklist) as described by Johnson et al. [34]; ** Chi Square test.

**Table 5:** The dietary habits scores calculated on the basis of the Adolescents’ Food Habits Checklist (AFHC) for purchase, preparation, consumption and total habits, within the PLACE-19 Study.

AFHC Scores *		Before the COVID-19 Pandemic	During the COVID-19 Pandemic	*p* ***	*r* ****
AFHC total score	Mean ± SD	12.72 ± 5.22	13.31 ± 5.40	0.001	−0.063
	Median (min–max)	12.65 ** (0.00–23.00)	13.32 ** (0.00–23.00)		
AFHC purchase score	Mean ± SD	2.88 ± 1.69	3.23 ± 1.66	<0.001	−0.120
	Median (min–max)	3.00 ** (0.00–6.00)	3.00 ** (0.00–6.00)		
AFHC preparation score	Mean ± SD	4.58 ± 1.98	4.69 ± 2.11	0.055	−0.032
	Median (min–max)	4.57 ** (0.00–8.00)	4.57 ** (0.00–8.00)		
AFHC consumption score	Mean ± SD	5.23 ± 2.43	5.38 ± 2.50	0.014	−0.040
	Median (min–max)	5.63 ** (0.00–9.00)	6.00 ** (0.00–9.00)		

* AFHC (Adolescents’ Food Habits Checklist) scores as described by Johnson et al. [34] for purchase, preparation, consumption and total habits; ** nonparametric distribution (assessed while using Shapiro-Wilk test; *p* ≤ 0.05); *** Mann-Whitney *U* test; **** rank-biserial correlation.

**Table 6:** The changes of dietary habits scores for purchase, preparation, consumption and total habits in categories of healthy, neutral and unhealthy habits calculated on the basis of the Adolescents’ Food Habits Checklist (AFHC) within the PLACE-19 Study.

	Habits		Measure of Habits			
	Before the COVID-19 Pandemic	During the COVID-19 Pandemic	AFHC Total Score	AFHC Purchase Score	AFHC Preparation Score	AFHC Consumption Score
Unchanged habits	Unhealthy	Unhealthy	476 (19.4%)	363 (14.8%)	452 (18.5%)	470 (19.2%)
	Neutral	Neutral	979 (40.0%)	829 (33.9%)	900 (36.8%)	931 (38.0%)
	Healthy	Healthy	473 (19.3%)	431 (17.6%)	425 (17.4%)	438 (17.9%)
	Total		1928 (78.7%)	1623 (66.3%)	1777 (72.7%)	1839 (75.1%)
Improved habits	Unhealthy	Neutral	122 (5.0%)	236 (9.6%)	150 (6.1%)	134 (5.5%)
	Neutral	Healthy	125 (5.1%)	168 (6.9%)	177 (7.2%)	166 (6.8%)
	Unhealthy	Healthy	14 (0.6%)	13 (0.5%)	10 (0.4%)	8 (0.3%)
	Total		261 (10.7%)	417 (17.0%)	337 (13.8%)	308 (12.6%)
Worsened habits	Neutral	Unhealthy	120 (4.9%)	227 (9.3%)	147 (6.0%)	127 (5.2%)
	Healthy	Neutral	123 (5.0%)	159 (6.5%)	174 (7.1%)	159 (6.5%)
	Healthy	Unhealthy	16 (0.7%)	22 (0.9%)	13 (0.5%)	15 (0.6%)
	Total		259 (10.6%)	408 (16.7%)	334 (13.6%)	301 (12.3%)
